# Supplementary material for: Fiscal policy and economic growth in Sub-Saharan Africa: Do governance indicators matter?
Source: PLoS One. 2023 Nov 27;18(11):e0293188. doi: 10.1371/journal.pone.0293188 (PMC10681179; doi:10.1371/journal.pone.0293188)
Supplement: S1 Table — (DOCX) [file pone.0293188.s001.docx]

**Sources and descriptions of the study variables**

| **Variables of the study** | **Description the variables** | **Source** |
| --- | --- | --- |
| Economic growth(GDPR) | GDP per capita is gross domestic product divided by midyear population. It is inflation adjusted GDP. | WDI |
| Government expenditure(GE) as a percentage of GDP | Includes all government current expenditures for purchases of goods and services (including compensation of employees). It also includes most expenditure on national defense and security, but excludes government military expenditures | WDI |
| Capital (GFCF) as percentage of GDP | Includes land improvements including schools, offices, hospitals, private residential dwellings, and commercial and industrial buildings. It is gross fixed capital formation. | WDI |
| Labor (LBF) total | Comprises people ages 15 and older who supply labor for the production of goods and services during a specified period. | WDI |
| Government effectiveness (goef) | Measures the quality of public services, the quality of the civil service and the quality of policy formulation and implementation, and the credibility of the government’s commitment to its stated policies. Scored as from -2.5 (less effective) to 2.5 (more effective). | WGI |
| Corruption control (cocor) | Control of Corruption index covers the idea of how public power is exercised for private gain. Scores range from − 2.5 to 2.5, with a higher value indicating that the perception of anti-corruption in the citizens is strong. | WGI |
| Voice and accountability(voac) | The voice and accountability index evaluates the level of freedom a country’s citizens have in the expression, association, and other media. It also presents how well citizens are able to participate in their government. Scores range from − 2.5 to 2.5, with higher score indicating a higher relative freedom of speech. | WGI |
| Regulatory quality(requ) | Captures perceptions of the ability of the government to formulate and implement sound policies and regulations that permit and promote private sector development. Performance score from 0 to 100. The highest score reflects the best situation. | WGI |

WDI denotes world development indicator, WGI world governance indicator
