# Supplementary material for: Fiscal policy and economic growth in Sub-Saharan Africa: Do governance indicators matter?
Source: PLoS One. 2023 Nov 27;18(11):e0293188. doi: 10.1371/journal.pone.0293188 (PMC10681179; doi:10.1371/journal.pone.0293188)
Supplement: Attachment — Submitted filename: Review Report-PONE-D-23-18802.docx [file pone.0293188.s002.docx]

# Title: Fiscal Policy and Economic Growth in Sub Saharan Africa: Do Governance Indicators Matter?

# Manuscript No.: PONE-D-23-18802

# Journal: PLOS ONE

# Recommendation: Major Revision

The abstract is well-structured and organized in a comprehensive manner.

While the introduction mentions the need for further study on the relationship between fiscal policy and economic growth in sub-Saharan Africa, it doesn't clearly highlight the specific research gap that the study aims to address. The introduction should provide a more focused and concise statement of the problem.

The introduction briefly mentions that the study employs the system GMM estimation method but does not provide a clear rationale for choosing this approach over alternative methods. It must be explained that why the chosen method is appropriate for investigating the research questions and how it addresses the limitations of previous studies.

The review does not explicitly mention the theoretical frameworks or concepts that underpin the studies reviewed. Including a theoretical foundation is important as it would support the framework for understanding the relationships between fiscal policy, governance, and economic growth.

The review focuses primarily on the impact of government expenditure on economic growth, with less emphasis on other aspects of fiscal policy or governance indicators. Provide a more balanced coverage of different dimensions of fiscal policy and governance to enrich the analysis.

Also this section could benefit from the inclusion of additional significant literary articles that could contribute further insights. Furthermore, there is a noticeable scarcity of cited literature pertaining this topic. Consequently, I recommend the incorporation of the following articles, as they align with the aim and objective of the current topic. It is important to cite these articles in your study for enhanced relevance and coverage:

<https://doi.org/10.1177/0972150919833484>

<https://doi.org/10.1108/JFC-01-2021-0003>

<https://doi.org/10.1177/0972150919846800>

<https://doi.org/10.1177/09721509221104849>

<https://doi.org/10.1016/j.gr.2023.05.007>

The significant to add Discussion section separately. Further, it is essential to engage in a comprehensive discussion of the findings. Merely interpreting the results is insufficient; authors should delve into the strategies, implications, and rationale underlying the derived outcomes.

To summarize, the conclusion of a study should be divided into three paragraphs: Conclusion, Practical Implications, and Limitations. The first paragraph should summarize the main points of the study, including its objectives, methodology, results, and overall interpretation of findings. The second paragraph should discuss the practical implications of the study and identify key beneficiaries such as policymakers, portfolio managers, the real estate industry, and others. It should also provide strategies for how these beneficiaries can apply the study's findings. The final paragraph should address the limitations of the study and offer suggestions for future researchers. The author should highlight potential areas of exploration that were not covered in the current study but could be explored by future scholars working on a similar topic.
