# Supplementary material for: Fiscal policy and economic growth in Sub-Saharan Africa: Do governance indicators matter?
Source: PLoS One. 2023 Nov 27;18(11):e0293188. doi: 10.1371/journal.pone.0293188 (PMC10681179; doi:10.1371/journal.pone.0293188)
Supplement: Attachment — Submitted filename: PLos response reveiwers comment.docx [file pone.0293188.s003.docx]

Date: 30^th^ July, 2023

**Response to Reviewers’ Comments**

**Manuscript No.:** PONE-D-23-18802

**Title:** Fiscal Policy and Economic Growth in Sub Saharan Africa: Do Governance Indicators Matter?

**Keywords:** Fiscal policy, Governance indicators, two-step system GMM, SSA countries

**Journal:** PLOS ONE

Dear Reviewers,

Greetings of the day!

We are thankful to the reviewers for taking the time to assess our manuscript, for their careful reading and for their suggestions and valuable comments which helped us to substantially improve the quality of our paper. In revising the manuscript, we have carefully considered all the raised comments and suggestions. We have attempted to succinctly explain the changes made in reaction to all comments. Our reply to each comment in point-by-point fashion is given in what follows

1. **On your concerns regarding abstract**

Respected reviewers, thank you for accepting the structure and organization of our abstract.

2. **On your concerns regarding introduction**

Dear respected reviewers, we have strongly and positively accepted your comments. Accordingly, we have thoroughly focused on the introduction section during review. We have now addressed the specific research gaps that the study aims to achieve. We have marked it in the marked up copy of our manuscript. Additionally, the introduction section is now focused. It concisely stated the problem of the study. The main reason of selecting system GMM is also discussed in the introduction section. Thank you for your insightful comments.

3. **On your concerns regarding literature review**

Dears, your concerns regarding theoretical literatures are well accepted. We have included theoretical reviews. Moreover, we have raised the evolution of fiscal policy from time of 1930 world great depression to the present institutionalism aspects of governance. We have enriched the both theoretical and empirical literature section.

Further, we have arranged literatures into two section; theoretical and empirical. Balanced coverage of different dimensions of fiscal policy and governance was provided. We have special regard to your supplementary literatures you supplied to us. We have also cited them in our work to enhance our manuscript.

4. **On your concerns regarding discussion section**

Dear reviewers, your concern regarding this section is also right. We appreciate and accepted it positively. Thus, we have added discussion section separately. We have also improved interpretation of the result of our study. In this section, we have provided sufficient discussion of the results.

5. **On your concerns regarding conclusion**

Following your good comments, the conclusion of a study is divided into three paragraphs. In the first paragraph we have captured conclusion while second paragraph dealt with the practical implications. Third paragraph of this section discussed limitation of the study and directions for future researches.

6. **Some Other parts of your concerns**

To sum up, we would like to appreciate your comments that made our manuscript smarter. Frankly, speaking we learned a lot. This is the way science and knowledge improves. We have included paragraph that discuss robustness check. Further, the major reason why government expenditure is conducted as a proxy of fiscal policy is explained and supported by literature. In developing countries, where the source of growth is mainly sourced from external debt not from tax revenue, the government expenditure can be considered as proxy of fiscal policy.

We would like to thank once again all the reviewers for taking the time to review our manuscript, for their relevant remarks and comments, and especially for their specifications which helped us to improve the quality of our paper.

Sincerely yours	Isubalew Daba
