# Supplementary material for: Fiscal policy and economic growth in Sub-Saharan Africa: Do governance indicators matter?
Source: PLoS One. 2023 Nov 27;18(11):e0293188. doi: 10.1371/journal.pone.0293188 (PMC10681179; doi:10.1371/journal.pone.0293188)
Supplement: Attachment — Submitted filename: Review Report-PONE-D-23-18802R1.docx [file pone.0293188.s004.docx]

# Title: Fiscal Policy and Economic Growth in Sub Saharan Africa: Do Governance Indicators Matter?

# Manuscript No: PONE-D-23-18802R1

# Journal: PLOS ONE

# Recommendation: Minor Revision

The author(s) should provide context and comprehensive background information to explain the study’s significance. Also, specify the scope of the study in terms of countries or regions covered and avoid unnecessary repetition of ideas.

In Presentation and discussion of the results (section 6), the results are just statistically interpreted. The author(s) need to illustrate, explain and justify the observed results by discussing the mechanism due which such impact is observed as well as support those results with past studies (the literature covered in Literature review section).

Discussion should be the end section but before Conclusion it should be placed. After applying all test like unit root, robustness checks and other, Discussion is added.

The sequence of the study is very confusing, author(s) are requested to follow the standard headings of the manuscript. Such as:

Chapter 1 Introduction

Chapter 2 Literature review

*Literature review can be divided into subheadings*

Chapter 3 Methodology and Data

Chapter 4 Data Analysis

*Discussion is part of Chapter 4*

Chapter 5 Conclusion

*The subheadings of this section are 5.1. Managerial Implications; 5.2. Future Recommendations*
