# Supplementary material for: Fiscal policy and economic growth in Sub-Saharan Africa: Do governance indicators matter?
Source: PLoS One. 2023 Nov 27;18(11):e0293188. doi: 10.1371/journal.pone.0293188 (PMC10681179; doi:10.1371/journal.pone.0293188)
Supplement: Attachment — Submitted filename: PLos response reveiwers comment R2.docx [file pone.0293188.s005.docx]

**Date: 7^th^ September, 2023**

**Response to Reviewers’ Comments**

**Manuscript No.: PONE-D-23-18802R1**

**Title:** Fiscal Policy and Economic Growth in Sub Saharan Africa: Do Governance Indicators Matter?

**Journal:** PLOS ONE

Dear Reviewers,

Greetings of the day!

We are thankful to both reviewers for taking the time to assess our manuscript, for their careful reading and for their suggestions and valuable comments which helped us to substantially improve the quality of our paper. In revising the manuscript, we have carefully considered all the raised comments and suggestions. We have attempted to succinctly explain the changes made in reaction to all comments. Our reply to each comment in point-by-point fashion is given in what follows.

1. **On your comments regarding significance and scope of the study**

Dear reviewers, thank you for raising this question. We have accepted it positively incorporated the issue. We have specified the scope of the study in terms of countries or regions. Moreover, we have now provided comprehensive background information to explain the study’s significance. We have highlighted this at the end of introduction (page 3 of the manuscript). We have also checked introduction section for unnecessary repetition of ideas.

2. **On your comments in presentation and discussion of the results (section 6 of previous version)**

Dear reviewers, thank you once again for the constructive comments you delivered to us. Frankly, our paper benefited a lot from this comment. In the revised manuscript, it is section 4. The discussion of the result is supported by the previous literatures. Discussion of the finding is beyond statistical interpretation of results (section 4.3 of the revised manuscript, page 18-22).

3. **On your comment on regarding sequence of the study**

Thank you so much once again for your comments. We have now followed the standard headings of the manuscript. At the end of the introduction, we have added this statement and we have arranged the sequence in line with your comments.

‘*The rest sections of the study are organized as follows. Section 2 presented literature review while methodology and data of the study are conferred in section 3. Further, Section 4 of this study provided data analysis whereas conclusions are discussed in section 5.’*

Moreover, we have included separate topic for discussion at end of section 4 but before conclusion.

We would like to thank once again all the reviewers for taking the time to review our manuscript, for their relevant remarks and comments, and especially for their specifications which helped us to improve the quality of our paper.

Sincerely yours, Isubalew Daba

Corresponding author
